# Naringenin Protects against Acute Pancreatitis in Two Experimental Models in Mice by NLRP3 and Nrf2/HO-1 Pathways

**DOI:** 10.1155/2018/3232491

**Published:** 2018-04-08

**Authors:** Yong Li, Yiyuan Pan, Lin Gao, Jingzhu Zhang, Xiaochun Xie, Zhihui Tong, Baiqiang Li, Gang Li, Guotao Lu, Weiqin Li

**Affiliations:** ^1^Surgical Intensive Care Unit (SICU), Department of General Surgery, Jinling Clinical Medical College of Nanjing Medical University, No. 305 Zhongshan East Road, Nanjing, Jiangsu Province 210002, China; ^2^Department of Gastroenterology, The Affiliated Hospital of Yangzhou University, Yangzhou University, Yangzhou, China

## Abstract

**Background:**

Naringenin (Nar) is a type of flavonoid and has been shown to have anti-inflammatory and antioxidative properties. However, the effects of Nar on acute pancreatitis (AP) have not been well studied. In this study, we aimed to investigate the function of Nar in a mouse model of AP.

**Methods:**

Mild acute pancreatitis (MAP) was induced by caerulein (Cae), and severe acute pancreatitis (SAP) was induced by L-arginine in mice. Nar was administered intraperitoneally at doses of 25, 50, or 100 mg/kg following MAP induction and at a dose of 100 mg/kg following SAP induction. The serum levels of cytokines, lipase, and amylase were determined, and pancreatic and pulmonary tissues were harvested.

**Results:**

The serum levels of amylase, lipase, and cytokines were significantly decreased in both MAP and SAP models after Nar treatment. The malondialdehyde (MDA) levels of the pancreatic tissue was significantly reduced in both MAP and SAP after Nar treatment. In contrast, glutathione peroxidase (GPx), glutathione reductase (GR), glutathione S-transferase (GST), total sulfhydryl (T-SH), and non-proteinsulthydryl (NP-SH) were markedly increased in both MAP and SAP after Nar treatment. The injury in pancreatic and pulmonary tissues was markedly improved as evidenced by the inhibited expression of myeloperoxidase, nod-like receptor protein 3, and interleukin 1 beta as well as the enhanced expression of nuclear factor erythroid 2-related factor 2/heme oxygenase-1 in pancreatic tissues.

**Conclusions:**

Nar exerted protective effects on Cae-induced MAP and L-arginine-induced SAP in mice, suggesting that Nar may be a potential therapeutic intervention for AP.

## 1. Introduction

Acute pancreatitis (AP) is an acute and life-threatening inflammatory disease that commonly damages peripancreatic tissues and other distant organs, leading to nearly 250,000 inpatient admissions at a cost of approximately $2.2 billion in the United States annually [1]. Pathophysiological characteristics of AP include local pancreatic tissue injury, systemic inflammatory responses, and multiorgan dysfunctions. Although most of the patients with AP have a mild course of the disease, 15% to 25% of patients with severe acute pancreatitis (SAP) develop into infected pancreatic necrosis and persistent organ failure [2], contributing mostly to AP mortality [3]. There is limited understanding of how the pancreatic acinar cell injury caused by the initial triggering events progresses into local tissue damage and even systemic inflammation. SAP, due to excessive release of inflammatory factors and increased oxidative stress response, can cause distant organ damage, especially acute lung injury. In addition, there is no effective therapeutic strategy for acute pancreatitis so far. It is well known that AP is a typical acute inflammatory response disease involving a variety of inflammatory cytokines, the activation of inflammasome, and the oxidative stress [4].

Naringenin (Nar) is a type of flavonoid, the predominant flavanone in grapefruit. Nar has been shown to have anti-inflammatory properties, organ-protective effects [5], and antioxidative functions [6]. Nar is involved in the regulation of many metabolic and signal transduction pathways such as the nuclear factor signaling pathway [7]. A recent study indicated that naringenin significantly protected against lipopolysaccharide-induced acute lung injury in rats [8]. Other studies have shown that oxidative stress plays a key role in the pathogenesis of acute pancreatitis induced by caerulein (Cae) [9, 10]. Free radicals have been found to participate in the development of the necrotic types of SAP induced by L-arginine (L-arg) [11]. Hence, in order to investigate the role of Nar in AP and the accompanying organ dysfunctions in mice as well as the underlying mechanisms, we used two animal models, Cae-induced mild acute pancreatitis (MAP) model and L-arg-induced SAP model. Moreover, we examined the features of pancreas and lung injury under the pathological condition.

## 2. Materials and Methods

### 2.1. Animals and Diets

Male mice in the Institute of Cancer Research (ICR) background weighing approximately 25–30 g were purchased from the Model Animal Research Center of Nanjing University (Nanjing, China) and were allowed to acclimatize for a minimum of 1 week prior to the experimentation. All mice were housed in a specific pathogen-free room under a 12/12 h light-dark cycle at 24°C with free access to water and fed standard laboratory chow. All the animal procedures were approved by the Animal Care and Use Committee of Nanjing University (number 20151008) and were carried out in accordance with the Guide for the Care and Use of Laboratory Animals issued by the National Institutes of Health.

### 2.2. Experimental Design and Procedures

In the Cae-induced MAP model, mice were randomly divided into 5 subgroups (*n* = 8–12 each group) as follows: control group, MAP model group, MAP + low-dose Nar (25 mg/kg) group, MAP + medium-dose Nar (50 mg/kg) group, and MAP + high-dose Nar (100 mg/kg) group. MAP was induced by 10 intraperitoneal injections of Cae (AnaSpec Inc., Fremont, USA) at a dose of 50 *μ*g/kg body weight (BW) in phosphate-buffered saline (PBS) at hourly intervals, and the control group was injected with PBS in the same way. In the Nar group, Nar (Sigma-Aldrich Chemical Co., St. Louis, MO, USA) was dissolved in 5% DMSO and injected intraperitoneally following AP induction immediately. The MAP model group was administered with the same volume of vehicle solution at the same time point as described above.

In the L-arg-induced SAP model, mice were randomly divided into 3 subgroups (*n* = 10 each group) as follows: control group, SAP model group, and SAP + Nar (100 mg/kg) group. SAP was induced by 2 intraperitoneal injections of 8% L-arg (pH = 7.4; Sigma-Aldrich Chemical Co., St. Louis, MO, USA) in PBS at a dose of 2 g/kg BW at hourly intervals, and the control group was injected with PBS in the same way. In the Nar (100 mg/kg) group, mice received intraperitoneal injections immediately following SAP model induction, and the SAP model group was administered with the same volume of vehicle solution at the same time point.

### 2.3. Biochemical Assays

Blood samples were obtained from the tail veins of sevoflurane-anesthetized mice at 0, 6, and 12 hours after the initial Cae injection and at 0, 24, 48, and 72 hours after initial L-arg injection. Blood samples were collected using heparinized syringes and centrifuged at 4000 rpm for 10 minutes at 4°C to separate the upper plasma from the lower cells for amylase and lipase measurements.

Amylase activity was measured using 5 ethylidene-G7PNP as a substrate with a commercial kit (Beijing Zhongsheng Beikong Biochemistry Company, China), and lipase activity was also measured with a commercial kit (Nanjing Jiancheng Biochemistry Company, China) according to the manufacturer's manual. The plasma levels of interleukin 1 beta (IL-1*β*), interleukin 6 (IL-6), and tumor necrosis factor alpha (TNF-*α*) were determined by enzyme-linked immunosorbent assay (ELISA) kits (eBioscience, San Diego, CA, USA) according to the manufacturer's manual.

### 2.4. Histological Examinations

Mice were anesthetized with an intraperitoneal administration of sodium pentobarbital (50 mg/kg) and then sacrificed. Pancreatic and pulmonary tissues were harvested and fixed in 4% paraformaldehyde and embedded in paraffin. Small parts of the pancreatic and pulmonary tissues were quickly frozen using liquid nitrogen and stored at −80°C until use.

The paraffin sections of the pancreas and lung tissue were stained with hematoxylin and eosin (H&E). Two pathologists who were blind to the experimental treatments evaluated the degrees of pancreatic injury by light microscopy by scoring the severity of edema, inflammation, and necrosis [12, 13]. We also evaluated the degree of pulmonary injury by scoring the severity of neutrophil infiltration, thickness of alveolar, and alveolar congestions [14, 15].

### 2.5. Immunofluorescence Examinations

Paraffin-embedded pancreatic and pulmonary tissue sections (5 *μ*m) were rehydrated in xylene and then in descending concentrations of ethanol solutions followed by high-temperature antigen retrieval in citrate buffer (10 mM, pH 6.0) for 20 minutes. Then these slides were cooled down at room temperature, rinsed with PBS, and treated with 0.3% H_2_O_2_ for 10 minutes to block endogenous peroxidase activity. Nonspecific binding was blocked with 10% goat serum albumin in PBS for 30 minutes at 37°C. Then these slides were incubated overnight at 4°C in a humidified chamber with rabbit polyclonal antibody anti-myeloperoxidase (MPO; 1 : 100 dilution) and incubated with fluorescein-labeled secondary antibody (1 : 200 dilution) for 1 h at room temperature. The slides were observed under a fluorescence microscope, and the photographs were captured using an Olympus CKX41 camera (Olympus Company, Japan). When evaluating MPO expression, ten fields across each slide were randomly selected for the analysis of the mean fluorescence intensity.

### 2.6. Measurement of Pancreatic MDA

Pancreatic tissue lipid peroxidation was determined by measuring MDA using thiobarbituric acid reactive substances. The pancreatic tissue was weighed and homogenized in potassium phosphate buffer (50 mmol/L, pH 7.4) containing butylated hydroxytoluene (12.6 mmol/L). Aliquots of the homogenate were incubated with thiobarbituric acid (0.37%) in an acidic solution (15% trichloroacetic acid and hydrochloric acid at 0.25 mol/L) at 90°C for 45 min. The homogenates were centrifuged (5 min, 8000 ×g), and aliquots from the supernatants were extracted using n-butanol, followed by vortexing for 30 s and centrifugation (2 min, 8000 ×g). The absorbance was measured at 535 nm in a microplate reader and calibrated at 572 nm. The results were calculated using a molar extinction coefficient of 1.55 × 10^5^ mol/L/cm and expressed as nmols of MDA per mg of tissue.

### 2.7. Measurement of Antioxidant Enzyme Activity

The GPx activity was determined as described previously [16]. The absorbance was monitored at 340 nm at 37°C for 10 min, and the results were expressed as *μ*mol of reduced glutathione (GSH)/min/mg of protein. The GR activity was measured as described previously [17] by measuring the consumption of nicotinamide adenine dinucleotide phosphate (NADPH) as a cofactor in the reduction of oxidized glutathione to reduced GSH. The results were expressed as U of GR/mg of protein. One U of enzyme activity was defined as the amount of GR that oxidizes 1 *μ*mol of NADPH per min. The GST activity was measured as described previously [18] using 1-chloro-2,4-dinitrobenzene (CDNB) as a substrate. The results were expressed as U of GST/mg of protein. One U of enzyme activity was defined as the amount of GST that produces 1 *μ*mol of the conjugate of GSH with CDNB per min. The total protein concentration in the homogenate was measured using the method of Bradford [19]. The levels of sulfhydryl compound, pancreatic T-SH, and NP-SH were determined by Ellman's reaction using 5′5′-dithio-bis-2-nitrobenzoic acid (DTNB). Aliquots of 4 mL homogenates in ice-cold ethylenediaminetetraacetic acid (0.02 mol/L; pH 8.9) were mixed with 3.2 mL of distilled water and 0.8 mL of 50% trichloroacetic acid. The tubes were centrifuged at 3000 ×g for 15 min. The supernatant (2 mL) was mixed with 4 mL Tris buffer (0.4 mol/L; pH 8.9) and 0.1 mL DTNB (0.01 mol/L). The absorbance was measured within 5 min after the addition of DTNB at 412 nm. The absorbance was extrapolated from a glutathione standard curve. Data were expressed as *μ*g/g of the tissue.

### 2.8. Western Blot Analysis

Pancreatic tissues were homogenized on ice and centrifuged at 4°C (13000*g*, 15 min). Then the cytoplasmic proteins in tissue homogenate were extracted using cytoplasmic extraction reagents (Pierce Biotechnology, Rockford, IL, USA) according to the manufacturer's instructions. The supernatants were collected, and the protein concentrations were determined. Equal amounts of protein (40 *μ*g/lane) were separated on 10% SDS-PAGE and transferred to nitrocellulose membranes. The membranes were blocked in 5% (*w*/*v*) skimmed milk and incubated with antibodies against mouse nod-like receptor protein 3 (NLRP3; 1 : 1000 dilution; Abcam, Cambridge, UK), IL-1*β* (1 : 1000 dilution; Cell Signaling Technology, Danvers, MA, USA), heme oxygenase-1 (HO-1; 1 : 1000 dilution; Abcam), nuclear factor erythroid 2-related factor 2 (Nrf2; 1 : 1000 dilution; Abcam), and *β*-actin (1 : 1000 dilution; Sigma-Aldrich Chemical Co.), followed by incubation with secondary goat anti-rabbit antibody (1 : 10,000 dilution) or secondary goat anti-mouse antibody (1 : 10,000 dilution) conjugated to horseradish peroxidase for 1 h at room temperature. The protein bands were quantified by the mean ratios of integral optical density normalized to the housekeeping gene *β*-actin expression.

### 2.9. Quantitative Reverse Transcription PCR (qRT-PCR)

The mRNA expression of NLRP3, IL-1*β*, Nrf2, and HO-1 in the pancreatic tissues of experimental mice was determined using qRT-PCR. Pancreatic tissues were homogenized in TRIzol® reagent (Life Technologies, Carlsbad, CA, USA), and total RNA was extracted according to the manufacturer's instructions. Then 6–8 ovaries from each group were transferred to 1.5 mL tubes and washed twice with RNase-free PBS. 350 mL of RNA extraction lysis buffer was added into each tube. The experiment was repeated three times. Total RNA was extracted using a RNA prep Pure Micro Kit (DP420; Tiangen Biotech, Beijing, China) according to the manufacturer's instructions. RNA concentrations were measured by a spectrophotometer (NanoDrop 2000c, Thermo Fisher Scientific, Waltham, MA, USA). Equal amounts of samples (100 ng/reaction) were reverse-transcribed using a FastQuant RT Kit (KR-106-02; Tiangen). A SYBR-based qPCR was then performed using Bestar qPCR Mastermix (DBI-2223; DBI Bioscience, Ludwigshafen, Germany) on an ABI StepOnePlus platform (Thermo Fisher Scientific). Quantitation of various mRNAs was performed, and GAPDH was used as the internal control. The relative mRNA expression was measured using the comparative 2^−(ΔΔCq)^ method. The primer sequences used to amplify mRNAs are shown in Table 1.

### 2.10. Statistical Analysis

Statistical analysis was performed using GraphPad Prism 6 software (GraphPad, San Diego, CA, USA), and data were presented as the mean ± standard deviation (SD). The results were analyzed using one-way analysis of variance, Student-Newman-Keuls test, and Mann–Whitney rank sum test. *P* < 0.05 was considered statistically significant.

## 3. Results

### 3.1. Naringenin Protected against Cae-Induced MAP

**Figure 1:**
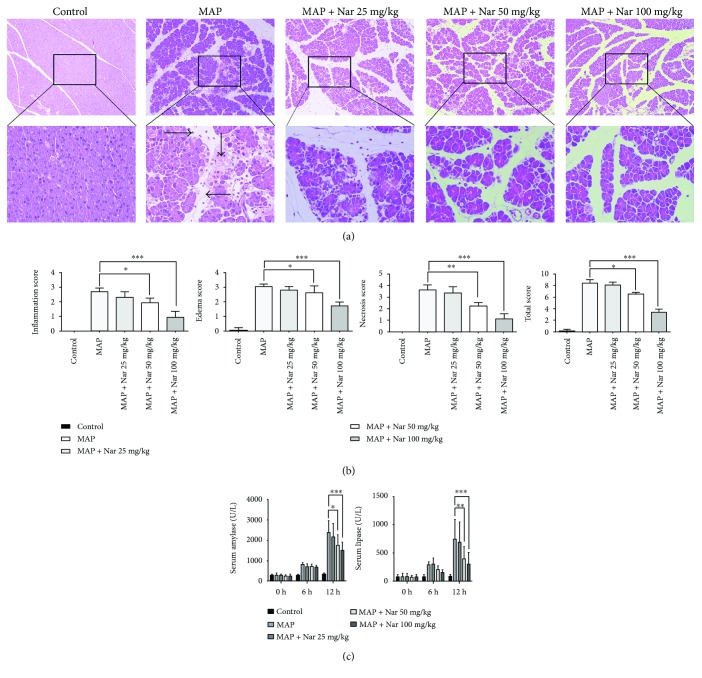
Naringenin alleviated the severity of pancreatic tissue injury in MAP. (a) Representative H&E staining of pancreatic tissues in magnifications 100x and 400x. (b) The pathological scores of pancreatic tissues. (c) Serum amylase and lipase levels of mice. ^∗^*P* < 0.05 compared with the MAP group. ^∗∗^*P* < 0.01 compared with the MAP group. ^∗∗∗^*P* < 0.001 compared with the MAP group. →: inflammation; ↓: acinar necrosis; ←: edema; MAP: mild acute pancreatitis; H&E: hematoxylin and eosin.

In our study, we found that Nar could alleviate the injuries of pancreatic tissues caused by Cae-induced pancreatitis in a dose-dependent manner. At the standard induction dose of Cae (50 *μ*g/kg), the pancreatic tissues were mainly characterized as obvious edema, inflammatory cell infiltration, and necrosis, while the pancreatic injuries in Nar-treated (100 mg/kg and 50 mg/kg) mice were significantly reduced compared with the MAP group (Figure 1(a)). In addition, the histological scores of pancreatic tissues in Nar-treated mice were remarkably lower than those in the MAP group (*P* < 0.001) (Figure 1(b)).

We also measured the serum parameters and found that, at the standard induction dose of Cae (50 *μ*g/kg), the serum amylase levels in the MAP group were remarkably higher than those in the MAP + high-dose Nar (100 mg/kg) group (*P* < 0.001) and MAP + low-dose Nar (50 mg/kg) group (*P* < 0.05). Similarly, the serum lipase levels in the MAP group were significantly higher than those in the MAP + high-dose Nar (100 mg/kg) group (*P* < 0.001) and MAP + low-dose Nar (50 mg/kg) group (*P* < 0.01) (Figure 1(c)).

### 3.2. Naringenin Protected against L-Arginine-Induced SAP

**Figure 2:**
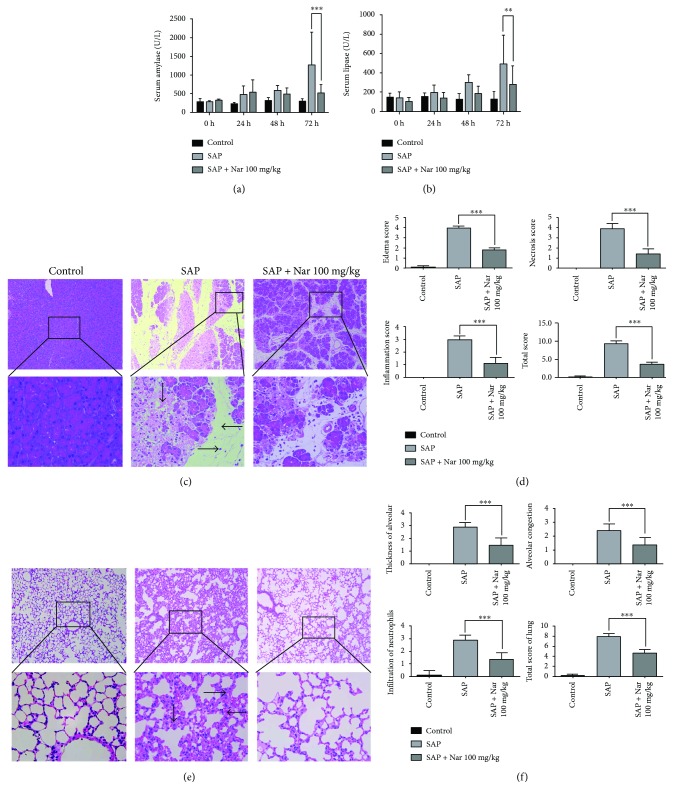
Naringenin alleviated the severity of pancreatic tissue injury in SAP. (a) Time course of serum amylase (a) and lipase (b) levels. (c) Representative H&E staining of pancreatic (c) and lung (e) tissues. Pathological scores of pancreatic (d) and lung (f) tissues. ^∗∗^*P* < 0.01 compared with the SAP group. ^∗∗∗^*P* < 0.001 compared with the SAP group. (c) →: inflammation; ↓: acinar necrosis; ←: edema. (e) ⟶: thickness of alveolar; ↓: infiltration of neutrophils; ⟵: alveolar congestion. SAP: severe acute pancreatitis; H&E: hematoxylin and eosin.

In the mouse model of Cae-induced AP, we found that Nar protected against AP in a dose-dependent manner. Hence, we selected 100 mg/kg as the intervention dose in the mouse model of L-arg-induced SAP and examined the serum levels of amylase and lipase. Consistently, the serum amylase and lipase levels were significantly higher in the SAP group than those in the SAP + high-dose Nar (100 mg/kg) group (*P* < 0.001) and SAP + low-dose Nar (50 mg/kg) group (*P* < 0.01) (Figures 2(a) and 2(b)).

In the mouse model of L-arg-induced SAP, we also found that pancreatic injuries in Nar- (100 mg/kg) treated mice were significantly alleviated compared with the SAP group. Accordingly, the histological scores were lower than those in the SAP group. These data indicate the protective roles of Nar in both MAP and SAP (Figures 2(c) and 2(d)).

Acute lung injury is one of the most prominent features of organ failures in SAP. According to the pulmonary H&E staining results, alveolar interval inflammatory cell infiltration and expansion of capillary congestion in Nar- (100 mg/kg) treated mice were significantly alleviated compared with those in the SAP group. Consistently, the histological scores of lung tissues in Nar-treated mice were significantly lower than those in the SAP group (*P* < 0.001) (Figures 2(e) and 2(f)).

### 3.3. Naringenin Reduced Inflammatory Cell Recruitment in Mouse Models of MAP and SAP

**Figure 3:**
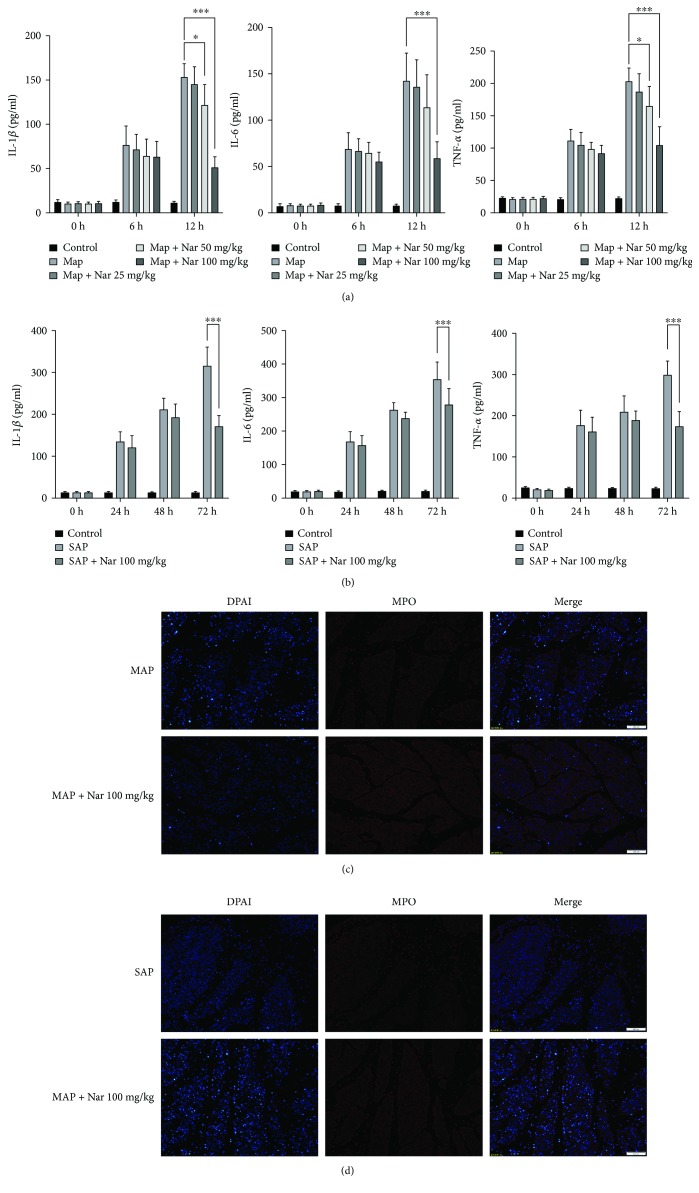
Naringenin inhibited the production of proinflammatory cytokines in MAP and SAP. (a) Serum levels of IL-6, IL-1*β*, and TNF-*α* in MAP (a) and SAP (b). Representative immunostaining of pancreatic MPO in MAP (c) and SAP (d) in magnification 40x. MAP: mild acute pancreatitis; SAP: sever acute pancreatitis; IL-6: interleukin 6; IL-1*β*: interleukin 1 beta; TNF-*α*: tumor necrosis factor alpha. ^∗^*P* < 0.05 compared with the MAP group. ^∗∗∗^*P* < 0.001 compared with the MAP group. ^∗∗∗^*P* < 0.001 compared with the SAP group.

In pancreatitis, the inflammatory response is triggered by the production of a series of inflammatory cytokines such as IL-6, IL-1*β*, and TNF-*α*. In the MAP model, the administration of a standard dose of Cae (50 *μ*g/kg) resulted in an elevation of serum IL-6, IL-1*β*, and TNF-*α* levels compared with the MAP group. In addition, Nar treatment (50 mg/kg) reduced all these parameters compared with the MAP group (*P* < 0.05) (Figure 3(a)). Interestingly, coadministration of Cae and Nar (100 mg/kg) reduced serum IL-6, Il-1*β*, and TNF-*α* levels even further (*P* < 0.001) (Figure 3(a)). The similar results were also observed in the SAP model (Figure 3(b)).

MPO is specifically expressed in neutrophils and is released into the circulation under the condition of inflammation. Therefore, MPO activity can reflect the activation of neutrophils. In this study, we performed immunofluorescent staining of MPO in the pancreatic tissues, which was used to reflect the degree of pancreatic inflammation. In the MAP group, the MPO staining of the pancreatic tissue was significantly stronger than that in the Nar- (100 mg/kg) treated group (Figure 3(c)). Nar also had the similar effect on MPO immunostaining in the SAP model (Figure 3(d)).

### 3.4. Naringenin Reduced the Pancreatic Generation of Oxygen-Free Radicals in MAP and SAP Mice

**Figure 4:**
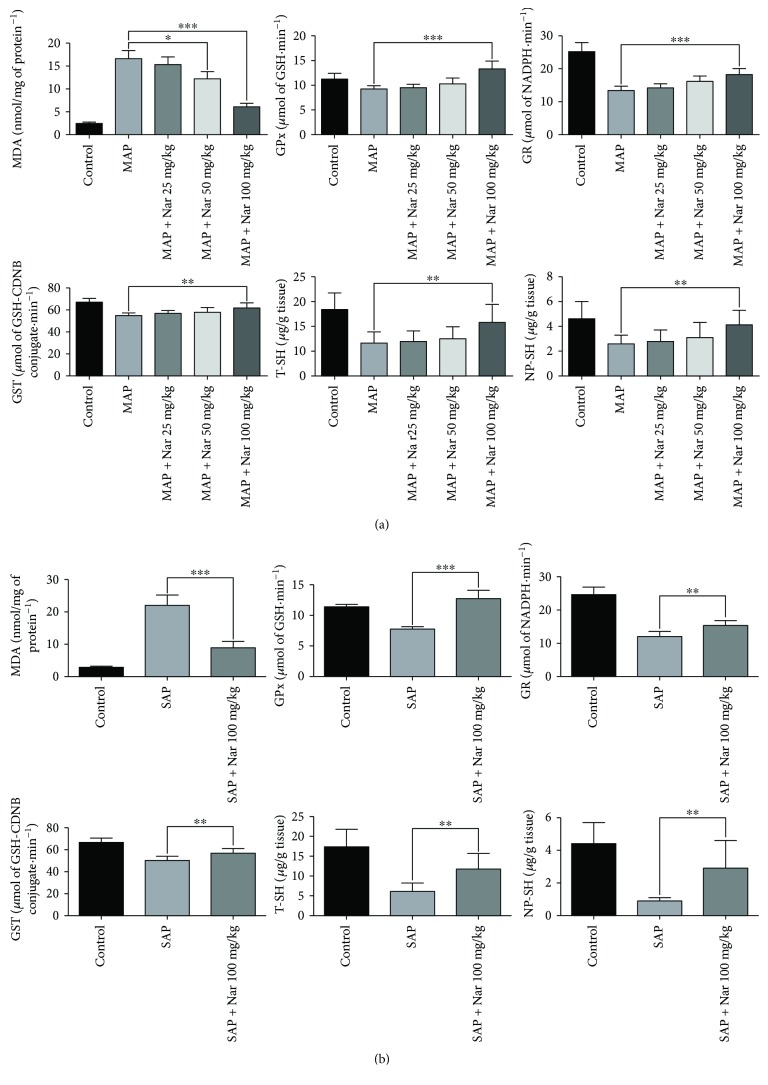
Naringenin inhibits oxidative stress in MAP and SAP. The levels of MDA, GPx, GR, GST, T-SH, and NP-SH were measured in pancreatic tissues in MAP (a) and SAP (b). ^∗^*P* < 0.05 compared with the MAP group. ^∗∗^*P* < 0.01 compared with the MAP or SAP group. ^∗∗∗^*P* < 0.001 compared with the MAP or SAP group.

Oxidative stress is involved in the inflammatory response of acute pancreatitis. We measured the levels of MDA, a lipid peroxidation marker, to reflect the degree of pancreatic injury. The results showed that Nar (100 mg/kg) treatment significantly reduced the MDA levels in the pancreatic tissue contrast with the MAP mice (*P* < 0.001) (Figure 4(a)). In the SAP + Nar 100 mg/kg group, the MDA levels in the pancreatic tissue were markedly decreased (Figure 4(b)). The GPx, GR, GST, TT-SH, and NP-SH were upregulated in both MAP and SAP after Nar treatment (100 mg/kg) (Figures 4(a) and 4(b)).

### 3.5. Naringenin Impaired NLRP3 Inflammasome Activation and IL-1*β* Production

**Figure 5:**
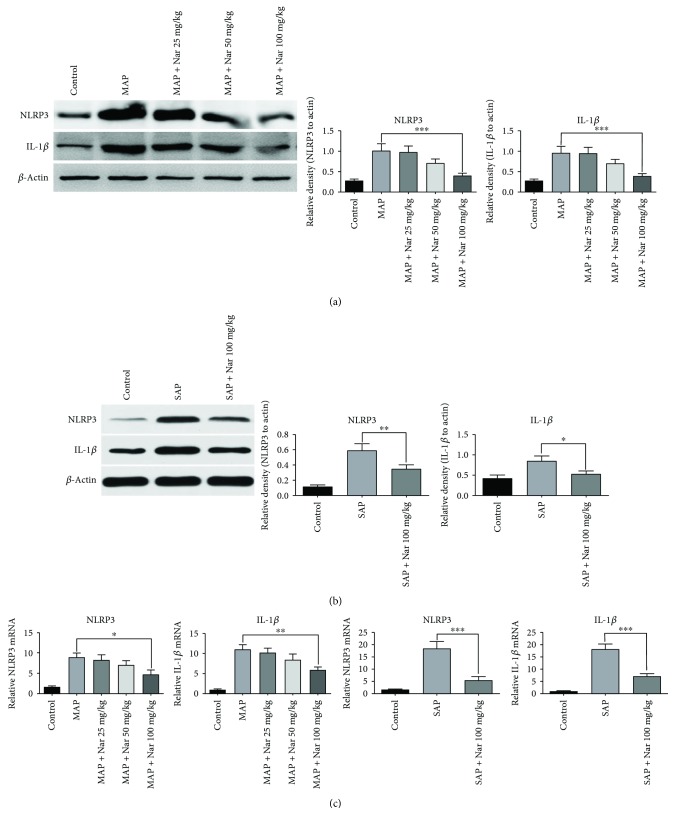
Naringenin suppressed expression of NLRP3 and IL-1*β* in MAP and SAP. Western blot analysis of NLRP3 and IL-1*β* protein expression in MAP (a) and SAP (b). (c) qRT-PCR analysis of NLRP3 and IL-1*β* mRNA expression in the pancreatic tissues in MAP and SAP. ^∗^*P* < 0.05 compared with the MAP or SAP group. ^∗∗^*P* < 0.01 compared with the MAP group. ^∗∗∗^*P* < 0.001 compared with the MAP or SAP group. NLRP3: nod-like receptor protein 3; IL-1*β*: interleukin 1 beta; MAP: mild acute pancreatitis; SAP: sever acute pancreatitis.

We performed Western blot analyses and qRT-PCR to detect the expression of NLRP3 and IL-1*β* in the pancreatic tissues in both models. Our results showed that the expression of NLRP3 was remarkably elevated in both models, and administration of Nar (100 mg/kg) significantly reduced the NLRP3 expression in pancreatic tissues compared with the MAP and SAP groups (*P* < 0.001 and *P* < 0.05, resp.) (Figures 5(a) and 5(b)). The activation of NLRP3 inflammasome promotes the maturation and release of IL-1*β*. Our results showed that the expression of IL-1*β* was remarkably inhibited in the Nar (100 mg/kg) pretreatment group compared with the MAP and SAP groups (*P* < 0.001 and *P* < 0.05, resp.) (Figures 5(a) and 5(b)). In addition, qRT-PCR was performed to detect the mRNA expression of NLRP3 and IL-1*β* in different groups. Our results indicated that Nar (100 mg/kg) treatment reduced both NLRP3 and IL-1*β* mRNA expressions in pancreatic tissues compared with the MAP group (*P* < 0.05 and *P* < 0.01, resp.) (Figure 5(c)). The similar results were also observed in the SAP model (Figure 5(c)).

### 3.6. Naringenin Enhanced Nrf2/HO-1 Expression in the Pancreatic Tissues in Both Models

**Figure 6:**
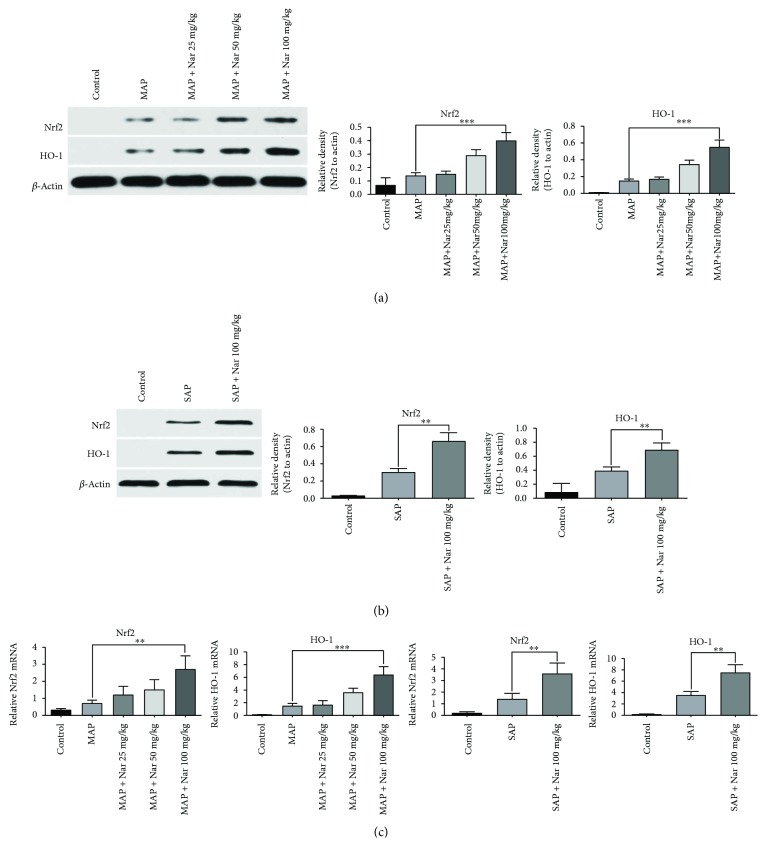
Naringenin enhanced protein expression of Nrf2 and HO-1 in MAP and SAP. Western blot analysis of Nrf2 and HO-1 expression in MAP (a) and SAP (b). (c) mRNA expression of Nrf2 and HO-1 in the pancreatic tissues in MAP and SAP by qRT-PCR. ^∗∗^*P* < 0.01 compared with the SAP group. ^∗∗∗^*P* < 0.001 compared with the MAP group. Nrf2: nuclear factor erythroid 2-related factor 2; HO-1: heme oxygenase-1; MAP: mild acute pancreatitis; SAP: sever acute pancreatitis.

Oxidative stress has been shown to play a vital role in the pathogenesis of AP, and the Nrf-2/HO-1 pathway is closely associated with oxidative stimulation. Nrf2 can translocate to the nucleus where it interacts with the antioxidant response element (ARE) to induce downstream gene expression, including HO-1. Previous studies indicated that the Nrf2/HO-1 pathway was majorly regulated in AP. In order to investigate whether Nar exerted antioxidant and anti-inflammatory effects on pancreatic tissues in the MAP and SAP models by inducing HO-1 and Nrf2 expression, we examined protein expression of HO-1 and Nrf2 by Western blotting and qRT-PCR following Nar treatment at different doses. The results demonstrated that Nar increased HO-1 protein level in a dose-dependent manner in the MAP model (Figure 6(a)). Similarly, Nar was also shown to induce HO-1 and Nrf2 expression at a high dose in the SAP model (Figure 6(b)). Collectively, these findings suggest that Nar plays a protective role against MAP and SAP possibly through the induction of Nrf2 and HO-1 (Figures 6(a)–6(c)).

## 4. Discussion

Our study has revealed that Nar exerted protective effects against both cerulean-induced and L-arginine-induced pancreatitis and distant organ injuries. Furthermore, we have confirmed that prophylactic administration of Nar could reduce pancreatic pathological injuries, inflammatory responses, and the activation of NLRP3 inflammasome as well as relieving the oxidative stress in experimental MAP and SAP mice. To our knowledge, we demonstrate for the first time that Nar may be used as a novel and effective therapeutic agent for AP.

As a type of flavonoid, Nar is the predominant flavanone in grapefruit and is found to have strong anti-inflammatory, antioxidant, and organ-protective activities. A previous study has indicated that Nar alleviates the histopathological changes in the liver and kidney caused by alloxan-induced diabetes in mice [20]. Nar can downregulate TNF-a, IL-1*β*, IL-6, IL-10, and other inflammatory cytokines in macrophages infected with live *Chlamydia trachomatis* [21]. Our study is consistent with these findings, and we also confirmed that administration of Nar could exert protective effects on both MAP and SAP. Nar has been shown to play protective roles even over a wide range of dosages or concentrations. Our study has further made sure that Nar pretreatment at 100 mg/kg has obvious protective effects. However, the potential role of Nar in inflammatory disease has not been extensively studied, especially in AP, which needs further investigation.

The inflammatory response is a hallmark in the pathogenesis and progression of pancreatitis and pancreatitis-induced distant organ injuries, in which the release of inflammatory cytokines and neutrophil exudation are two critical events. IL-6, IL-1*β*, and TNF-*α* are the most important cytokines involved in the inflammatory response, and their serum levels are directly associated with the severity of AP. In our study, we have concluded that Nar could alleviate the cascade activation of these inflammatory cytokines and generate the protective effects on organ injuries. MPO is mainly expressed in neutrophils and could serve as a biomarker of activated neutrophils. Our results were consistent with previous findings that MAP and SAP cause an enhancement in MPO expression in pancreatic tissues while Nar pretreatment leads to a significant decrease in MPO expression. Collectively, the cascade activation of inflammatory mediators and the overreaction of phagocytic cells along with their reciprocal interactions play important roles in the local tissue injury due to exaggerated inflammatory response. Nar appears to serve as a potential therapeutic agent for AP.

The inflammasome is a large multiprotein complex composed of nucleotide-binding domain and leucine-rich repeat-containing proteins or AIM2, adaptor protein ASC, and caspase-1 and plays a critical role in host defense against exogenous pathogens and inflammation [22, 23]. The canonical inflammasomes include NLRP3, NLRP1, NAIP–NLRC4, and AIM2. Among them, NLRP3 inflammasome is the most well-studied one and overactivation of the NLRP3 inflammasome is involved in the pathogenesis of several inflammatory diseases. A previous study indicates that inhibition of NLRP3 inflammasome activation attenuates experimental AP in mice [4]. Our results showed that NLRP3 inflammasome indeed played a vital role in AP and Nar could protect mice against MAP and SAP via inhibiting the activation of NLRP3 inflammasome.

The pathophysiology of AP is very complicated, and oxygen-derived free radicals are found to be involved in the pathogenesis of AP [24]. The pancreatic tissue is more susceptible to oxidative stress than other tissues because of extremely weak expression of antioxidative enzymes in pancreatic islet cells [25]. Oxygen free radicals generated during acute pancreatitis not only cause pancreatitis acinar cell damage but also contribute to the pancreatic damage [26, 27]. In terms of the anatomical location, physiological function, and hemodynamics, the pancreas and liver are closely related. The release of oxygen free radicals in AP reaches the liver through blood circulation and causes damage to the liver, resulting in a decreased ability of the liver to scavenge the free radicals and an increased systemic oxidative stress response. Previous findings have shown that in the rat model of Cae-induced AP, some substances such as tissue MDA are significantly upregulated. Therefore, we hypothesize that in mouse models of pancreatitis induced by Cae or L-arg, oxidative stress-related molecules like MDA released locally by the pancreatic tissue are transported to the liver, which impairs the liver's free radical-scavenging capacity, resulting in enhanced oxidative stress and pancreotoxic manifestations. The levels of indicators of oxidative stress correlate with the severity of acute pancreatitis. A recent study reveals that proanthocyanidin derived from grape seeds can function as a protective factor in the oxidative stress-mediated pancreatic dysfunction in rats via Nrf-2/HO-1 signaling [28]. The proanthocyanidin/Nrf-2/HO-1 axis also plays a key role in preventing oxidative stress in human bronchial epithelial BEAS-2B cells [29]. Another study has demonstrated that HO-1 and CO exert anti-inflammatory effects via decreasing the expressions of TNF-*α*, IL-1*β*, and macrophage inflammatory protein-1 and increasing IL-10 levels [30–32]. Based on these findings, we speculate that inhibition of oxidative stress is a major determinant in reducing the inflammation and decreasing the severity of pancreatitis. HO-1 can catalyze the degradation of heme to produce CO which acts as an antioxidant and is an important molecule in host defense against oxidative stress. It has anti-inflammatory abilities through downregulating the inflammatory factors such as matrix metalloproteinase 2 and COX-2 [33–35]. Nrf2 acts as an upstream regulator of ARE-dependent phase II enzyme, translocating to the nucleus where it interacts with the ARE to further promote the expression of antioxidant genes, including HO-1 [36, 37]. Our results indicated that Nrf2 was activated and HO-1 was upregulated in Nar-induced immune defense against oxidative stress. These findings provide a potential therapeutic strategy to prevent AP involving oxidative stress and exaggerated inflammatory responses.

In conclusion, Nar exerts protective effects on organ injuries of Cae- and L-arg-induced pancreatitis by inhibiting oxidative stress and inflammatory response via the inactivation of NLRP3 inflammasome and upregulation of Nrf2/HO-1 expression.

## Figures and Tables

**Table 1:** Primer sequences for RT-PCR.

Primer	Sequence (5′ to 3′)	
NLRP3	Forward	CGAGACCTCTGGGAAAAAGCT
	Reverse	GCATACCATAGAGGAATGTGATGTACA
IL-1*β*	Forward	TGTAATGAAAGACGGCACACC
	Reverse	TCTTCTTTGGGTATTGCTTGG
Nrf2	Forward	CAGTGCTCCTATGCGTGAA
	Reverse	GCGGCTTGAATGTTTGTC
HO-1	Forward	ACAGATGGCGTCACTTCG
	Reverse	TGAGGACCCACTGGAGGA
GAPDH	Forward	GGAGCGAGATCCCTCCAAAAT
	Reverse	GGCTGTTGTCATACTTCTCATGG
