# Corrigendum to “Naringenin Protects against Acute Pancreatitis in Two Experimental Models in Mice by NLRP3 and Nrf2/HO-1 Pathways”

**DOI:** 10.1155/2018/8621908

**Published:** 2018-07-29

**Authors:** Yong Li, Yiyuan Pan, Lin Gao, Jingzhu Zhang, Xiaochun Xie, Zhihui Tong, Baiqiang Li, Gang Li, Guotao Lu, Weiqin Li

**Affiliations:** ^1^Surgical Intensive Care Unit (SICU), Department of General Surgery, Jinling Clinical Medical College of Nanjing Medical University, No. 305 Zhongshan East Road, Nanjing, Jiangsu Province 210002, China; ^2^Department of Gastroenterology, The Affiliated Hospital of Yangzhou University, Yangzhou University, Yangzhou, China

In the article titled “Naringenin Protects against Acute Pancreatitis in Two Experimental Models in Mice by NLRP3 and Nrf2/HO-1 Pathways,” [1] there were errors in the SAP dosage administered to mice in the “Materials and Methods” and the “Results” sections.

In the Materials and Methods section, the text reading “SAP was induced by 2 intraperitoneal injections of 8% L-arg (pH = 7.4; Sigma-Aldrich Chemical Co., St. Louis, MO, USA) in PBS at a dose of 2 g/kg BW at hourly intervals, and the control group was injected with PBS in the same way” should be corrected to “SAP was induced by 3 intraperitoneal injections of 8% L-arg (pH = 7.4; Sigma-Aldrich Chemical Co., St. Louis, MO, USA) in PBS at a dose of 3 g/kg BW at hourly intervals, and the control group was injected with PBS in the same way.”

In the Results section and under the “Naringenin Protected against Cae-Induced MAP” subsection, the text reading “MAP + low-dose Nar (50 mg/kg) group” should be corrected to “MAP + medium-dose Nar (50 mg/kg) group.”

In the Results section and under the “Naringenin Protected against L-Arginine-Induced SAP” subsection, the text reading “and SAP + low-dose Nar (50 mg/kg) group (*P* < 0.01)” should be deleted.
